# Supplementary material for: Metagenomic analysis exploring taxonomic and functional diversity of soil microbial communities in Chilean vineyards and surrounding native forests
Source: PeerJ. 2017 Mar 30;5:e3098. doi: 10.7717/peerj.3098 (PMC5376117; doi:10.7717/peerj.3098)
Supplement: Supplemental Information 1 [file peerj-05-3098-s001.docx]

|  |  | Forest soil | | |  | Vineyard soil | | |
| --- | --- | --- | --- | --- | --- | --- | --- | --- |
| MG-RAST ID | | 4565463.3 | 4565459.3 | 4565458.2 |  | 4565462.3 | 4565460.3 | 4565461.3 |
| Sampling site | | Ocoa | Leyda | Apalta |  | Ocoa | Leyda | Apalta |
|  |  |  |  |  |  |  |  |  |
| Uploading information | |  |  |  |  |  |  |  |
|  | bp count | 78,236,955 | 61,669,011 | 87,262,168 |  | 94,090,245 | 84,497,008 | 86,785,396 |
|  | Sequences count | 178,272 | 141,694 | 195,138 |  | 208,095 | 189,372 | 201,266 |
|  | Mean sequence length (bp) | 438 ± 61 | 435 ± 62 | 447 ± 56 |  | 452 ± 49 | 446 ± 56 | 431 ± 65 |
|  | Mean GC content (%) | 63 ± 7 | 63 ± 7 | 63 ± 7 |  | 64 ± 7 | 63 ± 7 | 64 ± 7 |
| Post QC information | |  |  |  |  |  |  |  |
|  | bp count | 58,111,844 | 50,049,262 | 58,556,003 |  | 49,222,231 | 61,988,359 | 59,132,069 |
|  | Sequences count | 131,618 | 114,120 | 130,215 |  | 108,385 | 138,101 | 136,116 |
|  | Mean sequence length (bp) | 441 ± 60 | 438 ± 60 | 449 ± 54 |  | 454 ± 48 | 448 ± 54 | 434 ± 63 |
|  | Mean GC content (%) | 63 ± 7 | 63 ± 7 | 63 ± 7 |  | 64 ± 7 | 63 ± 7 | 63 ± 7 |
| Processed sequences | |  |  |  |  |  |  |  |
|  | Predicted protein features | 140,227 | 124,130 | 138,954 |  | 114,149 | 150,966 | 145,661 |
|  | Predicted rRNA features | 11,886 | 10,942 | 11,382 |  | 9,711 | 12,479 | 13,243 |
| Aligned sequences | |  |  |  |  |  |  |  |
|  | Identified protein features | 78,645 | 70,366 | 77,092 |  | 63,320 | 85,976 | 80,790 |
|  | Identified rRNA features | 100 | 107 | 94 |  | 92 | 137 | 117 |
| Annotated sequences | |  |  |  |  |  |  |  |
|  | Identified functional categories | 63,245 | 56,293 | 61,931 |  | 50,793 | 69,078 | 64,858 |
|  |  |  |  |  |  |  |  |  |
