# Supplementary figures and images for: Metagenomic analysis exploring taxonomic and functional diversity of soil microbial communities in Chilean vineyards and surrounding native forests
Source: PeerJ. 2017 Mar 30;5:e3098. doi: 10.7717/peerj.3098 (PMC5376117; doi:10.7717/peerj.3098)

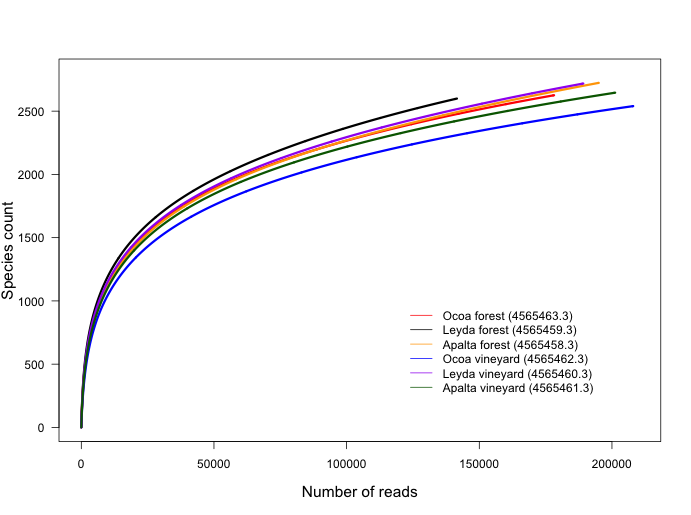

Supplement: Supplemental Information 2 — Rarefaction curves for sequences sampled in forest and vineyard soils. The accession numbers for the metagenomes in the MG-RAST server are indicated in parenthesis. [file peerj-05-3098-s002.docx]
